# Markers of inflammation are associated with clinical outcomes in patients with metastatic renal cell carcinoma treated with nivolumab

**DOI:** 10.1186/2051-1426-3-S2-P197

**Published:** 2015-11-04

**Authors:** Mario Sznol, Mayer Fishman, Bernard Escudier, David F McDermott, Harriet Kluger, Walter M Stadler, Jose Perez-Gracia, Douglas G McNeel, Brendan D Curti, Michael R Harrison, Elizabeth R Plimack, Leonard Appleman, Lawrence Fong, Charles G Drake, Tina C Young, Scott D Chasalow, Petra Ross-MacDonald, Jason S Simon, Dana Walker, Toni K Choueiri

**Affiliations:** 1 Yale Comprehensive Cancer Center, New Haven, CT, USA; 2 Department of Medical Oncology, H. Lee Moffitt Cancer Center and Research Institute, Tampa, FL, USA; 3 Department of Medical Oncology, Institut Gustave Roussy, Villejuif, France; 4 The Cytokine Working Group; Division of Hematology/Oncology, Beth Israel Deaconess Medical Center, Boston, MA, USA; 5 Department of Medical Oncology, Yale Cancer Center, New Haven, CT, USA; 6 University of Chicago Medicine, Chicago, IL, USA; 7 Department of Oncology, University Clinic of Navarra, Pamplona, Spain; 8 Department of Medicine, University of Wisconsin-Madison, Madison, WI, USA; 9 Providence Cancer Center, Providence Portland Medical Center, Portland, OR, USA; 10 Department of Surgery, Duke University Medical Center, Durham, NC, USA; 11 Department of Medical Oncology, Fox Chase Cancer Center, Philadelphia, PA, USA; 12 Department of Medicine, University of Pittsburgh Medical Center Cancer Pavilion, Pittsburgh, PA, USA; 13 Department of Medicine, University of California San Francisco Helen Diller Family Comprehensive Cancer Center, San Francisco, CA, USA; 14 Department of Oncology, Sidney Kimmel Comprehensive Cancer Center at Johns Hopkins University, Baltimore, MD, USA; 15 Bristol-Myers Squibb, Princeton, NJ, USA; 16 Department of Medicine, Dana-Farber Cancer Institute, Boston, MA, USA

## Background

In previously treated patients with metastatic renal cell carcinoma (mRCC), the programmed death-1 (PD-1) inhibitor antibody nivolumab demonstrated objective response rates of 20%–22% and median overall survival (OS) of 18.2– 25.5 months[1]. An exploratory biomarker analysis of baseline and on-therapy changes was conducted to investigate the relationship between the clinical and immunomodulatory activity of nivolumab.

## Methods

Patients with 1–3 prior therapies for mRCC received nivolumab 0.3, 2, or 10 mg/kg IV every 3 weeks (Q3W); treatment-naïve patients received 10 mg/kg IV Q3W. Biopsies and peripheral blood mononuclear cells were obtained at baseline and cycle 2 day 8. Tumor burden reduction was defined as a ≥20% decrease. Gene expression data were obtained on Affymetrix U219. OS parameters were estimated by the Kaplan-Meier method or by Cox proportional hazards regression. PD-1 ligand 1 (PD-L1) expression was measured by tumor membrane immunohistochemical staining (28-8 antibody; Dako) in baseline biopsies. Serum-soluble factors were quantified using a Luminex multiplex panel (Myriad Rules-Based Medicine). T cell receptor sequencing was conducted with the immunoSEQ assay (Adaptive Biotechnologies).

## Results

91 patients were treated. 59 baseline and 55 on-therapy biopsies were evaluable for gene expression, with 42 matched samples. Patients with tumor burden reduction had differential expression (>1.3-fold, *P* < 0.01, q-value < 0.16) of 311 genes at baseline (n = 13) and 779 genes on-therapy (n = 11) compared with patients without tumor burden reduction, including higher expression of transcripts associated with cell-mediated immunity. CTLA-4, TIGIT, and PD-L2 transcripts were present at higher levels on-therapy in patients with tumor burden reduction. Table 1 summarizes OS and OS by PD-L1 expression. 18/56 biopsies (32%) had ≥5% PD-L1 expression. Among serum-soluble factors, recognized prognostic markers (VEGF, ICAM1, VCAM1, TIMP1) were associated with OS. Based on T cell sequencing, increased tumor T cell counts and decreased blood T cell clonality at baseline were associated with longer OS.

**Table 1:** 

	Median OS, mo (95% CI)	OS rate, % (95% CI)	
		1-yr	2-yr
Treatment group			
0.3 mg/kg (n=22)	16.4 (10.1-NR)	71 (47-86)	44 (22-64)
2.0 mg/kg (n=22)	NR	72 (48-86)	61 (36-78)
10 mg/kg (n=23)	25.2 (12.0-NR)	74 (48-88)	51 (27-71)
10 mg/kg (naïve) (n=24)	NR	81 (57-92)	76 (51-89)

PD-L1 expression			
≥5% (n=18)	NR	71 (44-87)	64 (37-82)
<5% mg/kg (n=38)	23.4 (13.1-33.3)	71 (52-83)	48 (30-64)

NR = not reached

## Conclusions

Immune markers at baseline and on-therapy suggest pre-existing adaptive immunity is associated with nivolumab-induced tumor regression. Upregulation of immune checkpoint molecules provides rationale for study of nivolumab and ipilimumab combination in mRCC. A minimal difference in OS by PD-L1 expression was observed for up to 2 years.

## Trial registration

ClinicalTrials.gov identifier NCT01358721.
